# The dermatological manifestations and differential diagnosis of monkeypox: A narrative review

**DOI:** 10.1097/MD.0000000000040359

**Published:** 2024-11-01

**Authors:** Jacob Al-Dabbagh, Eman Mohammad Deeb, Razan Younis, Rahaf Eissa

**Affiliations:** a Cancer Research Center, Tishreen University, Latakia, Syria & Faculty of Medicine, Tishreen University, Latakia, Syria; b Faculty of Medicine, Tishreen University, Latakia, Syria; c Faculty of Medicine, Tartous University, Tartous, Syria.

**Keywords:** monkeypox, orthopoxvirus, outbreak, viral dermatosis, virus, zoonotic disease

## Abstract

Monkeypox (MPX) is a zoonotic viral disease caused by the monkeypox virus (MPXV), which belongs to the Orthopoxvirus genus. The main clinical features of MPX are fever, rash, and lymphadenopathy. It is usually a self-limited disease and can resolve within a few weeks in most cases. MPXV is now becoming a global concern. The world health organization declared the outbreak of MPX in 2022 a global health emergency. In this article, we focus on the mucocutaneous manifestations and differential diagnosis of MPX.

## 1. Introduction

Monkeypox (MPX) is a double-stranded DNA virus that belongs to the Orthopoxvirus genus of the Poxviridaem family.^[1,2]^ The main hosts of poxviruses are rabbits, rodents, and nonhuman primates.^[2]^ Other hosts, such as tree squirrels, rope squirrels, Gambian pouched rats, and dormice seemed to be the natural reservoirs of the MPX virus.^[3]^

Monkeypox virus (MPXV) is classified into 2 genetic clades, West African and Central African, each of which has a distinct epidemiology and symptomatology.^[4]^ According to reports, the Central African clade is more virulent and causes more severe forms of the disease.^[4]^

Transmission of MPXV both from animal-to-human and from human-to-human is well documented.^[5]^ MPXV can also be transmitted through fomites that are contaminated with this infection.^[6]^ It can be transmitted into the body through injured skin, the respiratory tract, or the mucous membranes (oral cavity, nose, or eyes).^[6]^ Some transmission examples include prolonged contact with respiratory secretions or saliva from an MPX patient, direct contact with skin lesions, vertical transmission, and consumption of meat from infected animals.^[3]^

MPX was first detected in a laboratory in Denmark in 1958 as an eruption of exanthema on the skin of imported monkeys.^[7]^ The first human-reported case of MPX was in August 1970 in the Democratic Republic of the Congo in a 9-month-old child.^[8,9]^ Since the 1970s, MPXV has led to several outbreaks, mostly in Central and West Africa.^[8]^ The MPX outbreak in 2003 in the United States was the first reported outbreak outside of Africa.^[8]^ For several years, there had been no reported cases of MPX until outbreaks of MPX occurred in Cameroon (2018) and Nigeria (2017–2021).^[8]^ In 2022, an unprecedented outbreak of MPXV was reported in several non-endemic countries and had a wide geographical spread.^[7,8]^ The number of cases continued to increase with the continued transmission; moreover, MPXV received new attention and is considered an international public health emergency.^[7,8]^

## 2. Systematic symptoms of monkeypox

The course of MPX infection can be classified into 4 stages: Incubation period, prodromal stage, rash stage, and crusting stage.^[10]^ World Health Organization (WHO) classifies 2 phases of MPXV infection: An initial invasive phase (a prodromal period) that can last up to 5 days and consists of fever (the most frequently reported prodromal symptom), headache, asthenia, malaise, myalgia, dorsalgia, and superficial lymphadenopathy, then a secondary skin rash phase that develops 1 to 3 days after the onset of the fever.^[1,10,11]^ When the patients are in the pustular period, a secondary fever may occur, indicating a worsening of their status.^[11]^ However, some pediatric patients may not experience fever.^[11]^

The incubation period of MPX is usually 6 to 13 days, but it may be prolonged up to 21 days.^[1,10]^ However, the incubation period in some cases was shorter, averaging 8.5 days, and the majority of these cases reported sexual contact, such as anal or oral sex, prior to the onset of the disease.^[10]^

Prodromal symptoms, such as fever and headache, were mild, and local rash at the site of contact was the initial symptom in some reported cases.^[10]^ Other symptoms such as cough, shortness of breath, nasal discharge, oral ulcers, sore throat, weight loss, abdominal pain, and diarrhea were reported too.^[9]^ Mucosal lesions, however, can be extremely painful and in severe cases might result in the perforation of the rectal wall.^[12]^

Localized enlargement of lymph nodes was noted in 85% of patients with cutaneous lesions, but not extensive lymph node enlargement^[10]^ However, asymptomatic infections were reported in some cases.^[12]^

## 3. Monkeypox risk factors and complications

MPX is self-limited in the majority of patients and can last between 2 and 4 weeks.^[5]^ The prognosis of MPX infection depends on several factors, including previous vaccination status, initial health status, co-occurring diseases, and comorbidities.^[1]^

Risk factors are associated with severe disease including immunocompromised patients (especially those with untreated or poorly managed concurrent HIV infection), invasive exposure, pediatric age group (younger than 8 years), pregnant women, and patients who have certain skin conditions (such as eczema).^[1,3,7,11]^

Complications are usually associated with the exposure severity to the virus and the patient’s health status.^[1]^

Secondary skin infections (such as abscess, furuncle, carbuncle, cellulitis, and necrotizing soft tissue infection) suppurative lymphadenitis, tonsillitis, posterior pharyngeal abscess, bronchopneumonia, respiratory distress, sepsis, septic shock, proctitis, encephalitis, myocarditis and corneal infections (which can lead to corneal scarring and permanent loss of vision) have been reported as severe symptoms and complications in some patients.^[1–3,5,11]^

Dehydration is one of the MPX complications that can be caused by painful oral enanthema resulting in dysphagia and decreased oral intake.^[13]^ Studies have also reported that infection with MPXV can cause neurological symptoms ranging from nonspecific symptoms such as headaches and myalgias to more uncommon symptoms such as encephalitis and seizures.^[14]^

According to estimates, the mortality rate from MPX infections in the general population of African countries ranges from 0% to 11%, with the highest mortality rate among young children.^[3]^

Encephalitis, sepsis, and acute respiratory distress syndrome are the main complications that lead to mortality in MPX patients.^[12]^

## 4. Mucocutaneous manifestations of monkeypox

In MPX patients, not all types of lesions may occur during an eruption, and not all lesions are at the same stage.^[3]^ Patients who have underlying immunodeficiency such as HIV infection and syphilis may present with atypical clinical manifestations and delay a proper diagnosis, increasing the risk of MPX transmission in healthcare facilities.^[15]^ The 2022 outbreak’s clinical characteristics differ in some respects from those of the earlier outbreaks.^[12]^ Compared with earlier outbreaks, the 2022 outbreak is characterized by fewer lesions occurring more frequently on mucosal surfaces.^[12]^ Moreover, during the 2022 MPX outbreak, patients with isolated lesions on the genital or perineal/perianal sites were reported with no skin involvement on any other sites.^[1]^ Anyhow, in earlier outbreaks, lesions occurred predominantly on the face and hands.^[12]^

Studies indicated that cutaneous lesions at sites of sexual contact suggest the possibility of sexual transmission.^[10]^ Oral lesions develop when oral sex occurs with a person who is already infected, whereas lesions on the face can result from rimming with an infected partner or simply from kissing.^[3]^ Genital lesions in male patients are frequently associated with surrounding edema, which can cause severe swelling of the glans or foreskin of the penis, preventing the retracted foreskin from returning to its normal position.^[16]^

The course of cutaneous manifestation of MPX can be divided into 2 stages: Rash stage and crust stage.^[10]^

### 4.1. Rash period

The rash has a very distinctive presentation and manifests as painful and/or pruritic maculopapular lesions that develop into a vesiculopustular rash, which is considered the characteristic feature of MPX.^[2,14]^

The rash usually occurs within 1 to 3 days after the onset of prodromal symptoms.^[2,5]^ However, the rash can occur more than 3 days after the fever or both the rash and the fever can occur simultaneously in some patients.^[2]^ The most common sites of skin lesions are the face, trunk, limbs, genitals, scalp, palms, and soles.^[10]^ The rash usually appears on the face then later becomes generalized by spreading centrifugally on the limbs, palms, and soles, as well as the oropharynx, conjunctival mucosa, cornea, and genitalia.^[1,5,10]^ The rash tends to be denser on the face and limbs (centrifugal distribution).^[11]^

According to the latest studies, MPXV appears to first infect the host through the mucous membranes before being transferred by Langerhans cells to the regional lymph nodes.^[17]^ After a short prodromal stage, during which the virus replicates in the lymphoid organs, the virus spreads via the blood, and consequently, prominent skin lesions typically occur only in specific skin areas.^[17]^

The typical rash manifests as macules and papules, measuring 0.5 to 1 cm in diameter, that gradually develop over a period of 14 to 21 days into vesicles and pustules (often with umbilication), and crusts.^[1,5]^ Vesicles and pustules are usually spherical, measuring 0.5 to 2 cm in diameter, with a hard texture, deep involvement, and distinct borders.^[10]^ The central depression, which resembles an umbilical fossa, can be accompanied by evident pain and itchiness.^[10]^

Patients may notice lesions developing on the tongue and in the mouth before the rash manifests on the skin; these lesions are described as enanthem.^[2]^ A recent scoping review article concluded that the most commonly reported oral symptom of MPX is sore throat, whereas the most frequent oral or circumoral sign is ulceration.^[18]^

The number of lesions varies from a few to thousands, and the seriousness of MPX can be classified into 4 levels based on the number of rashes: 1 to 25 as mild, 26 to 100 as moderate, 101 to 250 as grave, and more than 250 as plus grave.^[10]^ The rash gradually develops, and lesions that have the same size or at the same sites are usually at the same stage of development.^[1,10]^ Each stage lasts 1 to 2 days and the pustular period can last 5 to 7 days.^[10]^ However, in the 2022 outbreak, atypical lesions were described at various stages of development.^[1]^

Some children first develop 1 or more rashes in the genital area and/or around the anus, which are followed by fever and enlarged lymph nodes.^[11]^ In some severe cases, the rash can coalesce and even large pieces of the skin can peel off.^[11]^

### 4.2. Crusting period

In a period of 2 to 4 weeks, the rash resolves into crusting and desquamation.^[1]^ The peeled crusts may be much smaller in size compared to the original lesion.^[10]^ When the crusts drop off, pitted scars, erythema, or areas of hyper- and hypo-pigmentation may persist.^[2,10]^ Pitted scars are the most frequent long-term complication in pediatric patients.^[11]^

**Figure 1.**
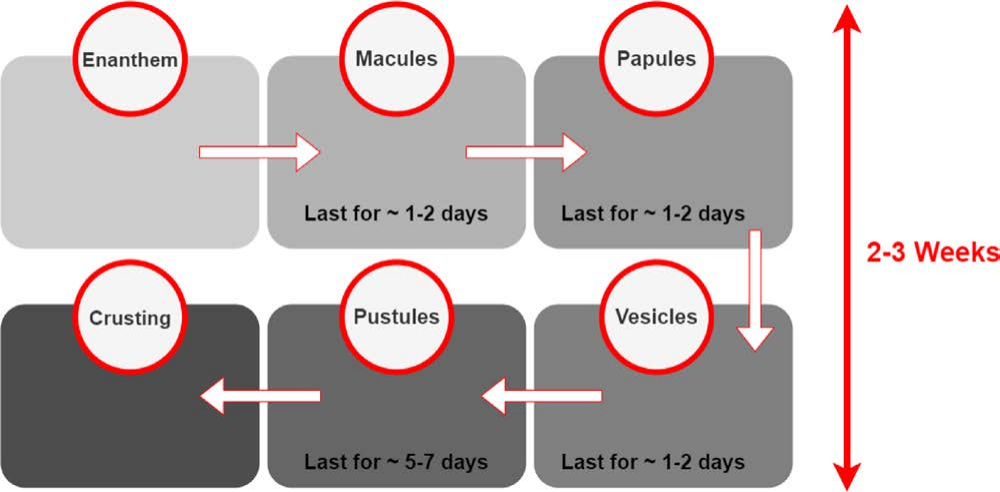
The periods of monkeypox in the rash stage.

The cutaneous lesions are painful at all stages of the vesiculopustular rash in MPX patients until the desquamation stage.^[2]^ Furthermore, pain caused by MPX is a frequent hospitalization reason.^[10]^ The crusting can cause extreme itching in the affected person.^[2]^ The periods of the MPX rash are illustrated in Figure 1.^[2]^

Typically, the onset of a rash indicates the commencement of the infectious period and is contagious until the crusts drop off.^[10]^ Prodromal periods can also be contagious, according to the Centers for Disease Control and Prevention (CDC).^[10]^ However, after the crusts drop off, the patients are no longer contagious.^[4]^

Secondary skin infection is the most frequent complication of MPX.^[10]^ Extensive cutaneous damage can also lead to secondary bacterial infection, which results in extensive scarring.^[10]^ Involvement of the oral mucosa can result in difficulty in eating and drinking, with eventual oral ulceration, pharyngitis, tonsillitis, and epiglottitis.^[10]^

## 5. Diagnosis

According to WHO recommendations, any person presenting with a suspected case, or a person of any age who presents in a non-MPX-endemic country with an unexplained acute rash and with 1 or more of the following signs or symptoms should be tested for the presence of MPX.^[7]^ Headache, asthenia, a sudden increase in fever over 38.5°C, myalgia, back pain, and lymphadenopathy should be investigated.^[7]^

MPX should be highly suspected in individuals with skin lesions and other risk factors like contact history (such as sexual contact and contact sports), travel from endemic regions (such as Africa), and men who have sex with men (MSM).^[12]^

The presence of typical mucosal and/or skin lesions, systemic symptoms, and/or potential contact with an MPX-infected person within the past few weeks are the criteria for the diagnosis of MPX.^[1]^ The diagnosis should also be suspected in people who present with genital ulcer or proctitis that does not respond to empirical treatment.^[5]^

Diagnostic tests include virus isolation in cultures of mammalian cells in specialized laboratories, electron microscopy, conventional or real-time polymerase chain reaction (PCR), enzyme-linked immunosorbent assay (ELISA), and immunofluorescent antibody assay.^[1,19]^

The preferred diagnostic laboratory test of MPX is conventional or real-time PCR with high accuracy and sensitivity, according to WHO, the PCR test is the only used method and the gold standard for MPX diagnosis.^[1,17]^ Samples from cutaneous lesions are most suitable to perform PCR tests to diagnose MPX.^[1]^ Samples should be obtained with swabs of vesicles, pustules (from the roof or fluid), or dry scales gathered in different tubes.^[1]^ It is recommended to collect samples from at least 2 or 3 skin lesions at different sites and in different stages of evolution.^[1,5]^ In addition to samples from cutaneous lesions, swabs can also be taken from the oropharynx.^[1]^

Blood samples are considered unsuitable for PCR diagnosis of MPX because of the time-limited viremia, which affects sampling after the presence of symptoms.^[1]^ However, studies have shown that MXPV can be detected in saliva, semen, urine, and feces.^[1,5]^

Currently, WHO recommends nucleic acid amplification tests for the detection of the MPX genome of the virus.^[20]^ In addition, ELISA can be used to detect specific IgM and IgG antibodies in the serum of MPX-infected patients at 5 to 8 days postinfection.^[9]^

## 6. Differential diagnosis of the monkeypox dermatological manifestations

WHO lists measles, chickenpox, smallpox, bacterial dermatosis, syphilis, and drug eruption as differential diagnoses of MPX.^[10]^

When cutaneous lesions appear, a thorough examination of the distribution and morphologic features of the rash, as well as a concomitant whole-body examination, are required.^[11]^ Initially, MPX should be distinguished from other infectious diseases that can cause cutaneous lesions, such as smallpox, chickenpox, tanapox, herpes simplex, herpetic whitlow, herpes zoster, Orf, hand-foot-mouth disease (HFMD), dengue fever, scabies, and syphilis.^[5,11,19]^ Additionally, it should be distinguished from noninfectious diseases such as allergic diseases (contact dermatitis, fixed drug eruption, severe drug eruption, and papular urticaria), inflammatory diseases, allergic purpura, bullous pemphigoid, and neoplastic diseases (cutaneous mast cell hyperplasia and bullous Langerhans cell histiocytosis).^[11]^ Also, umbilicated vesicopustules in MPX may be confused with translucent umbilicated papules of genital molluscum contagiosum (MC), especially in patients with lesions restricted to the genitals.^[5]^ Lymphadenopathy, which often occurs early along with fever, is a characteristic feature of MPX compared to other diseases that look similar initially such as smallpox, chickenpox, and measles.^[4,21]^

### 6.1. Chickenpox and herpes zoster

Chickenpox, also known as varicella, and herpes zoster are caused by varicella-zoster virus (VZV) which is a highly contagious virus that enters the body through the respiratory tract.^[22]^ VZV can also spread via fomites from varicella and shingles skin lesions.^[22]^

Chickenpox, especially in severe cases, is the most common misdiagnosis of MPX.^[4,10]^ Both MPX and chickenpox occur commonly in childhood.^[11,23]^ Also, they are anatomically similar in the macule and papule presentations in their early stages.^[18]^ The chickenpox rash begins on the chest and back and then spreads to the body, while the MPX rash first appears on the face and then spreads centrifugally over the body.^[2,23]^

The location of the vesicles is the most important for distinguishing secondary VZV infections from MPXV infections because they are unilateral in the former and do not have a distinct pattern in the latter.^[18]^

The absence of lymphadenopathy is a useful indicator to distinguish VZV from MPX, which develops in MPX but not in VZV infections^[3,18]^

While MPX patients have fever 1 to 4 days before the rash onset, chickenpox patients have no fever or have fever a day before the rash onset.^[3,18]^

### 6.2. Smallpox

The clinical symptoms of MPX and smallpox are quite similar, and their clinical manifestations are difficult to differentiate due to the similar prodromal period, onset, and distribution of the rash.^[10]^ In addition, both of the diseases can form pitted scars on the skin after the crusts fall off.^[2,4,10]^ Nonetheless, smallpox and MPX can be differentiated by the existence of lymphadenopathy in MPX, which is absent in smallpox.^[4]^

Compared to smallpox, which has a 30% mortality rate, MPX has a lower mortality rate (about 10%).^[2,4]^

### 6.3. Measles

Measles and MPX are very similar diseases.^[24]^ Both of them are airborne, commonly occur in children, and cause comparable symptoms.^[11,24,25]^

Around 10 days after measles infection, the first symptoms appear in the form of fever and cough, and the patient becomes extremely contagious.^[25]^ At this time, ulcerated white lesions are formed on the buccal mucosa, which are known as Koplik spots.^[25]^ Then, a blotchy red rash appears on the face at the hairline and spreads over the rest of the body downwards.^[25,26]^

### 6.4. Herpes simplex virus

Herpes simplex virus (HSV) infection is transmitted primarily through contact with mucous membranes, skin, or mucosal secretions from individuals who are actively infected with HSV.^[27]^ HSV can also be transmitted by respiratory droplets from an asymptomatic patient.^[27]^

Children by the age of 5 are mainly infected with the orolabial HSV-1.^[27]^ Whereas HSV-2 is primarily sexually transmitted and therefore less common in young children.^[27]^

The prevalence of HSV is higher in women than in men, in contrast to MPX, which is most common in gay and bisexual men between 20 and 50 years.^[18,27]^

In terms of oral presentation, the 2 types of HSV (HSV-1 and HSV-2) may be similar to the oral presentation of MPX.^[18]^

Unlike MPX, which tends to affect adults, primary herpetic gingivostomatitis occurs commonly in children, with lesions similar to those of MPX.^[18]^ Whereas, secondary herpetic gingivostomatitis always occurs in adults, which makes MPX diagnosis more challenging.^[18]^

Lesions in secondary herpetic gingivostomatitis are restricted to keratinized, attached mucosa, while oral lesions in MPX are widespread.^[18]^

### 6.5. Hand-foot-and-mouth disease

Both HFMD and MPX mainly affect children.^[11,28]^ HFMD can be transmitted via nasal and throat secretions, blister fluid, or stool from infected people.^[28]^ It has a short incubation period (3–6 days) compared to MPX.^[2,28]^ The HFMD lesions typically develop on the oral cavity, including the buccal mucosa, hard palate, cheek surface, gums, and tongue, as well as the palms of the hands and soles of the feet.^[28]^ The MPX rash lasts about 14 to 28 days, compared to 7 to 10 days for HFMD.^[18]^ Unlike HFMD, lymphadenopathy is common in MPX.^[18]^

HFMD is usually self-limiting with a small proportion of children experiencing severe complications.^[28]^ On the other hand, MPX is mostly self-limiting with deaths occurring mostly in children due to complications.^[11]^

### 6.6. Cowpox

Cowpox can be acquired in humans by implanting a virus into injured skin after having contact with infected animals, generally cats or rats.^[29]^ Unlike MPXV, no human-to-human transmission has been reported.^[11,29]^ Cowpox lesions initially appear as erythematous macules that progress to papules and seropapules, followed by ulcerated plaques and eschar formation.^[29]^ MPX and cowpox are both self-limiting diseases and can produce scarring after healing.^[11,29]^

### 6.7. Herpangina

Herpangina, like MPX, is self-limiting and affects mostly children.^[11,18,30]^ It is hallmarked by enanthem presenting as discrete erythematous macules that develop into vesicles and eventually a central ulcer in the oral cavity.^[18]^ Similar to herpangina, MPX develops also enanthem which is painful in both diseases.^[12,18]^ However, herpangina develops no skin lesions.^[18]^

### 6.8. Orf

Both human-to-human and animal-to-human transmissions are documented in the Orf virus and MPXV.^[11,31]^ The primary Orf lesion is a small, firm, red-to-blue papule at the site of the Orf virus entrance, characteristically on a finger or hand.^[31]^ The papule progresses into a hemorrhagic pustule or bulla, which can form a central crust that may bleed.^[31]^ However, the lesions may present as a generalized vesiculopapular rash on the skin and mucous membranes.^[31]^ In addition, Orf lesions may be accompanied by low-grade fever, usually lasting only a few days, and moderate lymphadenopathy.^[31]^

### 6.9. Epstein-Barr virus

Epstein-Barr virus (EBV), unlike MPXV, is observed most frequently in adolescents but has common transmission methods and similar systematic symptoms to MPXV.^[3,4,10,11,32]^

The oral lesions of EBV are painless and only occur on the border of the tongue, whereas MPX oral lesions are painful and usually affect the tip and ventral/dorsal surfaces of the tongue.^[18]^

### 6.10. Syphilis

Syphilis is a widespread infection with increasing incidence, especially among MSM, but the systemic and cutaneous lesions do not appear until months after the canker sore.^[33]^ Syphilis, similar to MPX, can be transmitted vertically or by sexual contact.^[4,12,34]^

The signs and symptoms of MPX are comparable to those of secondary syphilis, including rash, fever, headache, pharyngitis, and lymphadenopathy.^[18]^ The progression of the rash in secondary syphilis may be popular, annular, or pustular with a gray pseudomembrane.^[18]^ However, the development of the rash in MPX follows a different pattern.^[18]^

Oropharyngeal syphilis with a painless tonsillar ulcer and progressive cervical lymph node enlargement is considered a differential diagnosis of oropharyngeal MPXV.^[33]^

### 6.11. Disseminated gonococcal infection

Disseminated gonococcal infection commonly affects women and presents as characteristic skin lesions initiated as pinpoint erythematous macules that develop into vesiculopustular, tender, or necrotic papules, or hemorrhagic bullae.^[35]^

While the majority of disseminated gonococcal infection cases have fewer than 25 or 30 total lesions, The number of MPX lesions varies from several to thousands.^[10,35]^

### 6.12. Chancroid

Chancroid typically presents, about 4 to 7 days following sexual contact, as painful erythematous papules at the sites of microabrasion which often develop into pustules and then open ulcers over several days.^[36]^

While chancroid is common in heterosexual men, MPXV commonly affects bisexual and homosexual men.^[18,36]^

### 6.13. Lymphogranuloma venereum

Classically, the lymphogranuloma venereum (LGV) course comprises of 3 separate stages.^[37]^ The first stage of LGV starts with a small, painless papule or pustule that may develop into a small, herpetiform ulcer.^[38]^ The lesion is localized at the bacterial inoculation site and may also occur in the mouth or throat.^[38]^

The second stage is associated with local lymphadenopathy which is usually unilateral.^[38]^ These LGV stages have common features with MPX.^[1,5,10,37,38]^

### 6.14. Granuloma inguinale

Granuloma inguinale, like MPX, can be transmitted through sexual contact and vertical transmission.^[4,12,37]^ It presents as solitary or multiple papules or nodules that may later develop into a painless ulcer and spread to adjacent tissues.^[37]^ Lymphadenopathy is a characteristic feature of MPX, whereas the lymph nodes are rarely affected in granuloma inguinale.^[10,37]^

### 6.15. Molluscum contagiosum

MC lesions can appear as multiple or single lesions which are typically present as umbilical, white, pink, or flesh-colored papules or nodules.^[39]^ The main affected sites in children are exposed skin areas, whereas lesions are most commonly found in the lower abdomen, thighs, genitals, and perianal region in adults.^[39]^

MC virus is transmitted vertically or through direct, sexual, nonsexual contact with infected skin, or autoinoculation.^[39]^ Infected fomites can also transmit the disease.^[39]^ Children are the most affected age group in both MPX and MC cases.^[11,39]^

### 6.16. COVID-19

COVID-19, similar to MPX, can be transmitted by close contact through respiratory droplets, direct contact with infected individuals, or contact with infected fomites.^[4,40]^ However, MPXV does not spread over a long-range airborne transmission such as COVID-19.^[3]^ Some COVID-19 patients can be contagious during their incubation period whereas the incubation period in MPX is not contagious.^[2,41]^ COVID-19 may rarely be present with a pustulovesicular eruption and should be considered as a differential diagnosis of MPX.^[5]^

### 6.17. Scabies

Scabies, like MPX, can also transmitted by skin-to-skin contact or by fomites.^[4,42]^ The characteristic distribution of scabies infestation includes the areas between the fingers, wrists, axillae, umbilicus, thighs, groin, genitals, buttocks, and the breasts (in females).^[42,43]^ The most frequently affected areas in infants and young children are the palms, soles, and the head.^[42,43]^ The prevalent symptom of scabies infection is severe and persistent itching.^[42]^

### 6.18. Scarlet fever

Scarlet fever is manifested by a diffuse bright red rash and significant desquamation after the rash resolves.^[44]^ Children are the most vulnerable age groups in both scarlet fever and MPX cases.^[11,44]^

Similar to MPX, scarlet fever can be transmitted through direct contact and through infected saliva or nasal secretions.^[4,18]^

The most common oral symptom of scarlet fever is a strawberry tongue which characterized by hyperplastic fungiform papillae coated in white.^[18]^ However, oral scarlet fever is not usually associated with ulcerated lesions.^[18]^ On the other hand, oral thrush in MPX may be accompanied by a rash.^[18]^

Scarlet fever requires antibiotic therapy; otherwise, the disease may relapse or progress into rheumatic fever after 1 to 5 weeks, with poststreptococcal glomerulonephritis a possible side effect.^[18]^ On the contrary, moderate MPX symptoms may resolve with or without supportive therapy.^[18]^

A summary of the causative organism, age prevalence, incubation period, the most involved sites, and typical clinical manifestations of the differential diagnosis of MPX is demonstrated in Table 1.^[4,18,23–31,34–39,42,43,45–59]^

**Table 1:** The differential diagnoses of cutaneous manifestations of monkeypox.

Differential diagnosis	Causative organism	Age prevalence	Incubation period	The most involved sites	The typical clinical manifestations
Chickenpox (Varicella)	Varicella-zoster virus	Children	11 to 21 d	The rash initially appears on the chest and back then spreads on the face, scalp, and extremities. Buccal mucosa, palate, tongue, gingival mucosa, and oropharynx are typical locations of intraoral vesicles.	The lesions develop into small papules and then vesicles. Over the next few days, the vesicles rupture and then crust over. Then, new lesions develop in crops over a period of multiple days.
Smallpox	Variola virus	Young age groups	9 to 11 d	The lesions have a centrifugal distribution and start on the forearms or face then 1 to 3 d later spread to the rest of the body.	Characteristic rash that develops in various stages (red spots in the mucous membrane and skin – raised papules – fluid-filled umbilical vesicles – pustules – crust – scab).
Measles	Measles virus	Young children	10 to 14 d	The rashes begin on the face (at the hairline) and extend downward to the rest of the body.	Red blotchy rashes. Ulcerated white lesions, called pathognomonic Koplik patches, occur on the buccal mucosa.
Herpes simplex type 1	Herpes simplex virus type 1 (HSV-1)	Children and adults	Around 4 d, but can range from 2 to 12 d	Mouth, pharynx, face, and eye.	Small monomorphic vesicles on an erythematous base erupt into painful, shallow, gray ulcers or erosions with or without crust formation.
Herpes simplex virus (HSV) genitalis	Herpes simplex virus type 1 and 2 (HSV-1–2)	It varies greatly by country, region within a country, and population subgroup.	2 to 12 d	Anogenital region. In men: Penile shaft and glans. In women: Perineum, labia majora, and labia minora. Perianal lesions in men and women.	Vesicles or ulcers.
Herpes zoster	Reactivation of varicella-zoster virus (VZV)	Elderly individuals	2 wk	The trigeminal (cranial), cervical, and thoracic sensory nerves.	A painful unilateral rash (macules, papules, and vesicular).
Hand-foot-mouth disease	Coxsackievirus A16 (CV-A16) and enterovirus 71 (EVA71)	Children under the age of 10	3 to 6 d	Oral cavity, palms, and soles.	Exanthema begins as a papule accompanied by blisters that eventually progress to an ulcer.
Cowpox	Cowpox virus	Young age groups	8 to 12 d	Hands, fingers, face, and neck.	A characteristic rash that develops in various Stages (macules with yellow, pink, or red coloration – papules – fluid-filled vesicles – pustules – hemorrhagic ulcerated lesions). These lesions usually form crusts or scabs that fall off, leaving scars.
Herpangina	Coxsackie virus (22 enterovirus serotypes)	Children	5 to 7 d	The soft palate, the buccal wall, posterior pharynx, the posterior third of the tongue, and tonsils.	Discrete erythematous macules that develop into vesicles, and eventually a central ulcer.
Orf	Parapoxvirus	Adults	About 1 wk	The dorsal aspect of hands and fingers.	The lesion evolves through 6 stages: Maculopapular stage (erythematous macule or Papule); the target stage (necrotic center and red outer halo); acute stage (the nodule starts to weep); the regenerative stage (the nodule becomes dry); papilloma stage (the lesion has become papilloma-like and forms a dry crust); the regression stage (the skin recovers its normal appearance, often without scar residue).
Epstein-Barr virus	Epstein-Barr virus (EBV)	Adults	About 6 wk	On the lateral border of the tongue.	Oral hairy leukoplakia.
Syphilis	Spirochetal Gram-negative bacterium Treponema pallidum	Adults	Primary syphilis: 2 to 6 wk Secondary syphilis: 4 to 10 wk	Primary syphilis: Penis (in men), cervix and labia (in women), and the anal canal, rectum, or oral mucosa (in both sexes). Secondary syphilis: A localized or generalized skin rash.	Primary syphilis: Indurated solitary ulcer. Secondary syphilis: Mucocutaneous papules coalesce into pinkish-gray lesions.
Disseminated gonococcal infection	Neisseria gonorrhoeae	Young adults	2 to 3 wk	Superficial mucous membranes.	Abscess, ulcer, or folliculitis.
Chancroid	Bacteria Haemophilus ducreyi	Adults	3 to 7 d (rarely up to 10 d)	In males: Distal penis. In females: Vulva and cervix. Perianal area in both sexes.	The lesion manifests as papules which develop into pustules and then frank ulcers.
Lymphogranuloma venereum	Chlamydia trachomatis	Adults over 24 yr	3 to 30 d	In women: vulva or posterior wall of vagina or cervix. In men: Penis.	A small papule or pustule may develop into a small and herpetiform ulcer.
Granuloma inguinale (donovanosis)	Klebsiella granulomatis comb.nov. (formerly Calymmatobacterium granulomatis)	Old adults between the ages of 20 and 40	About 50 d	Genital area.	Single or several papules or nodules later evolve and grow into a painless ulcer that may spread to adjacent tissues.
Molluscum contagiosum	Molluscum contagiosum virus	Children under the age of 14	From 1 to several weeks up to 6 mo	In children: Exposed skin areas, genitals, and face. In adults: The lower abdomen, thighs, perianal area, and genitals.	Umbilical pink- or skin-colored papules.
COVID-19	Coronaviruses	No age prevalence	1 to 14 d	Trunk	Vesiculobullous eruptions such as vesicles and bullous.
Scabies	Sarcoptes scabiei mite	1 to 4 yr old children (in tropical and low/middle-income countries).	2 to 6 wk (in cases of mainly infestation). 1 to 3 d (in cases of reinfestation).	Areas between the fingers, wrists, axillae, groins, genitals, buttocks, and breasts (in women). In infants and young children: Palms, soles, and head.	Itchy skin eruption consists of papules, nodules, vesicles, and burrows.
Scarlet fever	Gram-positive A beta-hemolytic streptococci group	Children aged 5 to 15	2 to 10 d	Tongue	The strawberry tongue.

## 7. Prevention

To minimize the risk of exposure and infection, people should try to avoid unprotected contact with wild animals, particularly sick or dead animals, including their meat and blood.^[11]^ All foods containing animal meat or organs must be properly cooked before being consumed.^[11]^

To prevent transmission of MPXV infection in the community, it is recommended that patients with confirmed or suspected MPX with mild or/and uncomplicated disease be isolated during the period of infection if the home environment assessment determines infection prevention criteria (IPC) are met in the home environment.^[7]^

If appropriate isolation and IPC procedures cannot be provided at home, then isolation may need to be provided in a healthcare facility or other designated facility with the informed consent of the patient and the agreement of the caregiver and the members of the household.^[7]^

CDC suggests the following procedures to minimize the risk of contracting MPX: Avoiding close skin and sexual contact with people who have a MPX-like rash; avoiding contact with materials and objects used by someone who has MPX; washing hands frequently; and using an alcohol-based hand sanitizer before touching the face and before eating.^[3,7]^

## 8. Treatment

At present, the best way to treat MPX is still unknown.^[1]^ There are no specific treatment options for MPX.^[2]^ To guarantee the best possible information, the United States food and drug administration has created a dedicated MPX website.^[1]^ Clinical care of MPX should be fully optimized to manage and prevent complications and long-term sequelae.^[20]^

The general treatment strategies for MPX cases include proper rest, adequate calorie and fluid intake, maintenance of the water-electrolyte balance and homeostasis, and monitoring of vital signs.^[11]^ Signs related to children’s mental state and nutrition, such as lethargy, irritability, poor spirits, and pallor, should be closely monitored.^[11]^ Patients should be also treated for any secondary bacterial infections.^[20]^

Antiviral drugs are promising treatment options.^[1]^ Tecovirimat, cidofovir, brincidofovir, vaccinia vaccine, and vaccinia immune globulin are suggested to treat MPX.^[1,2]^ Anyhow, most patients are treated symptomatically, and only a few may require specific antiviral treatment.^[5]^

Common complications requiring treatment in patients with atypical MPX include local abscesses, proctitis, tonsillitis, and penile edema.^[10]^ Additionally, in patients with severe infection or at risk of severe infection, antiviral treatment should be considered.^[1]^ Those may include patients with serious medical conditions (such as patients requiring hospitalization), people who are at risk for serious illness (such as immunocompromised individuals, children, particularly < 8 years of age, pregnant women, and individuals with cutaneous diseases and/or abnormal local infections).^[1]^

## 9. Conclusion

The outbreak of monkeypox disease in non-endemic areas is a reminder that infectious diseases and pathogens are not limited by geographic borders. Since the disease manifests mainly dermatologically, knowledge of clinical presentation and differential diagnosis is fundamental for dermatologists.

All individuals in the high-risk population with blisters or pustular eruptions and lymphadenopathy must be examined early, and similar skin disorders must be excluded. It is important to provide suitable supportive care to minimize the risk of complications as much as possible.

## Author contributions

**Conceptualization:** Jacob Al-Dabbagh.

**Supervision:** Jacob Al-Dabbagh.

**Validation:** Eman Mohammad Deeb, Razan Younis, Rahaf Eissa.

**Writing – original draft:** Jacob Al-Dabbagh.

**Writing – review & editing:** Jacob Al-Dabbagh, Eman Mohammad Deeb, Razan Younis, Rahaf Eissa.
